# SIFlore, a dataset of geographical distribution of vascular plants covering five centuries of knowledge in France: Results of a collaborative project coordinated by the Federation of the National Botanical Conservatories

**DOI:** 10.3897/phytokeys.56.5723

**Published:** 2015-09-29

**Authors:** Anaïs Just, Johan Gourvil, Jérôme Millet, Vincent Boullet, Thomas Milon, Isabelle Mandon, Bruno Dutrève

**Affiliations:** 1 Fédération des Conservatoires Botaniques Nationaux, 18 rue Beaumarchais 93100 Montreuil; 2 Conservatoire botanique national du Massif central Le Bourg 43230 Chavaniac-Lafayette France

**Keywords:** Metropolitan France, La Reunion Island, Plantae, Tracheophyta, FCBN, CBN

## Abstract

More than 20 years ago, the French Muséum National d’Histoire Naturelle^1^
(MNHN, Secretariat of the Fauna and Flora) published the first part of an atlas of the flora of France at a 20km spatial resolution, accounting for 645 taxa (Dupont 1990). Since then, at the national level, there has not been any work on this scale relating to flora distribution, despite the obvious need for a better understanding. In 2011, in response to this need, the Federation des Conservatoires Botaniques Nationaux^2^
(FCBN, http://www.fcbn.fr) launched an ambitious collaborative project involving eleven national botanical conservatories of France. The project aims to establish a formal procedure and standardized system for data hosting, aggregation and publication for four areas: flora, fungi, vegetation and habitats. In 2014, the first phase of the project led to the development of the national flora dataset: SIFlore. As it includes about 21 million records of flora occurrences, this is currently the most comprehensive dataset on the distribution of vascular plants (Tracheophyta) in the French territory. SIFlore contains information for about 15'454 plant taxa occurrences (indigenous and alien taxa) in metropolitan France and Reunion Island, from 1545 until 2014. The data records were originally collated from inventories, checklists, literature and herbarium records. SIFlore was developed by assembling flora datasets from the regional to the national level. At the regional level, source records are managed by the national botanical conservatories that are responsible for flora data collection and validation.

French Muséum National d’Histoire Naturelle^1^

Federation des Conservatoires Botaniques Nationaux^2^

In order to present our results, a geoportal was developed by the Fédération des conservatoires botaniques nationaux that allows the SIFlore dataset to be publically viewed. This portal is available at: http://siflore.fcbn.fr. As the FCBN belongs to the Information System for Nature and Landscapes’ (SINP), a governmental program, the dataset is also accessible through the websites of the National Inventory of Natural Heritage (http://www.inpn.fr) and the Global Biodiversity Information Facility (http://www.gbif.fr). SIFlore is regularly updated with additional data records. It is also planned to expand the scope of the dataset to include information about taxon biology, phenology, ecology, chorology, frequency, conservation status and seed banks.

Information System for Nature and Landscapes’

A map showing an estimation of the dataset completeness (based on Jackknife 1 estimator) is presented and included as a numerical appendix.

**Purpose**:

SIFlore aims to make the data of the flora of France available at the national level for conservation, policy management and scientific research. Such a dataset will provide enough information to allow for macro-ecological reviews of species distribution patterns and, coupled with climatic or topographic datasets, the identification of determinants of these patterns. This dataset can be considered as the primary indicator of the current state of knowledge of flora distribution across France. At a policy level, and in the context of global warming, this should promote the adoption of new measures aiming to improve and intensify flora conservation and surveys.

## Data published through

FCBN: http://www.siflore.fcbn.fr

## Introduction

### Taxonomic coverage

Note: The taxonomic and nomenclatural reference for the first edition of the SIFlore dataset is the fifth edition of the taxonomic repository for the fauna, flora and fungi of metropolitan France and overseas territories, TAXREF, which was developed within the framework of a convention between the French ministry of ecology, the MNHN, the FCBN and Tela Botanica. The overall methodological framework at the basis of the TAXREF repository is presented in Gargominy et al. (2014).

The version originally used for data aggregation is TAXREF v5.0, posted online on July 20th, 2012.

At the time of writing, the current version of TAXREF was v8.0, posted online on December 1st, 2014. Data is available in the taxonomic and nomenclatural reference TAXREF v5.0 on the http://siflore.fcbn.fr web atlas. For practical reasons, data has been automatically linked to TAXREF v8.0 on the GBIF website, which may generate some taxonomic errors. This work was carried out under the responsibility of the Service du patrimoine naturel (SPN/MNHN). In order to prevent any error of interpretation, amended taxa were tagged in the Darwin core archive within the field datageneralization as follows: “taxon initially attached to the Taxonomic repository TAXREF v5. This taxon has undergone changes since”.

It has to be noted that the taxonomic coverage of La Reunion Island was incomplete in this 1^st^ version of SIFlore dataset as TAXREF v5.0 did not include most of La Reunion Island taxa. This issue was adressed in TAXREF v7, and subsequent versions, with the integration of the “ Index des Trachéophytes de La Réunion (ITR, Boullet & al, 2012) ”. Consequently, SIFlore completeness will be improved in the near future by including more taxa originating from La Réunion.

**Figure 1.**
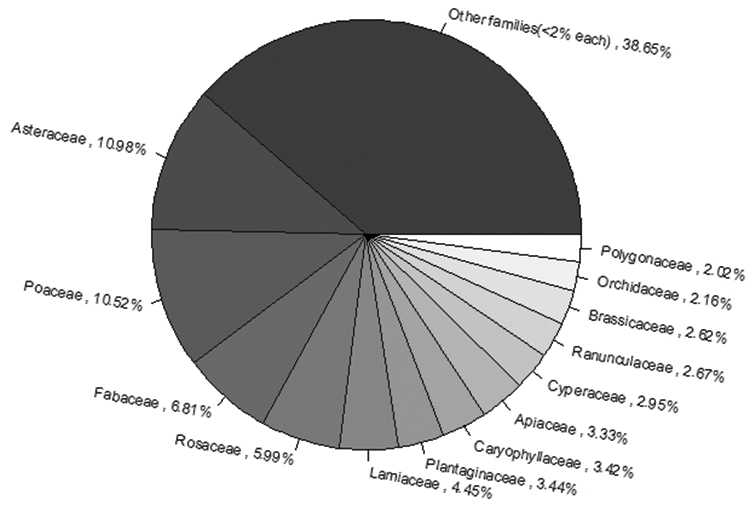
Distribution of the dataset by family.

**General taxonomic coverage description**: The taxonomic coverage of this dataset spans the phylum Tracheophyta (vascular plants) present in metropolitan France (excluding the departments of Alsace and Lorraine) and La Reunion Island. Taxa are first identified at the species level and, if appropriate, subspecies level. The largest number of data records belong to the Asteraceae family (2,317,247 records), followed by Poaceae (2,220,479 records), Fabaceae (1,436,758 records) and Rosaceae (1,264,321 records). The families with the least number of records are Escalloniaceae, Malpighiaceae and Schizaeaceae with one data record each (Figure 1).

## Taxonomic ranks

**Kingdom**: Plantae

**Phylum**: Tracheophyta

**Class**: Equisetopsida

**Order**: Acorales, Alismatales, Apiales, Aquifoliales, Araucariales, Arecales, Asparagales, Asterales, Boraginales, Brassicales, Buxales, Caryophyllales, Celastrales, Ceratophyllales, Commelinales, Cornales, Crossosomatales, Cucurbitales, Cupressales, Dioscoreales, Dipsacales, Ephedrales, Equisetales, Ericales, Escalloniales, Fabales, Fagales, Garryales, Gentianales, Geraniales, Ginkgoales, Gunnerales, Hymenophyllales, Isoetales, Lamiales, Laurales, Liliales, Lycopodiales, Magnoliales, Malpighiales, Malvales, Myrtales, Nymphaeales, Ophioglossales, Osmundales, Oxalidales, Pandanales, Pinales, Piperales, Poales, Polypodiales, Proteales, Psilotales, Ranunculales, Rosales, Salviniales, Santalales, Sapindales, Saxifragales, Schizaeales, Selaginellales, Solanales, Vitales, Zingiberales, Zygophyllales

**Family**: Acanthaceae, Acoraceae, Actinidiaceae, Adoxaceae, Aizoaceae, Alismataceae, Altingiaceae, Amaranthaceae, Amaryllidaceae, Anacardiaceae, Annonaceae, Apiaceae, Apocynaceae, Aponogetonaceae, Aquifoliaceae, Araceae, Araliaceae, Araucariaceae, Arecaceae, Aristolochiaceae, Asparagaceae, Aspleniaceae, Asteraceae, Balsaminaceae, Basellaceae, Begoniaceae, Berberidaceae, Betulaceae, Bignoniaceae, Blechnaceae, Boraginaceae, Brassicaceae, Bromeliaceae, Butomaceae, Buxaceae, Cabombaceae, Cactaceae, Campanulaceae, Cannabaceae, Cannaceae, Capparaceae, Caprifoliaceae, Caricaceae, Caryophyllaceae, Casuarinaceae, Celastraceae, Ceratophyllaceae, Cistaceae, Cleomaceae, Clusiaceae, Colchicaceae, Combretaceae, Commelinaceae, Convolvulaceae, Coriariaceae, Cornaceae, Crassulaceae, Cucurbitaceae, Cunoniaceae, Cupressaceae, Cymodoceaceae, Cyperaceae, Cytinaceae, Dennstaedtiaceae, Dioscoreaceae, Droseraceae, Dryopteridaceae, Ebenaceae, Elaeagnaceae, Elatinaceae, Ephedraceae, Equisetaceae, Ericaceae, Escalloniaceae, Euphorbiaceae, Fabaceae, Fagaceae, Frankeniaceae, Garryaceae, Gentianaceae, Geraniaceae, Gesneriaceae, Ginkgoaceae, Goodeniaceae, Grossulariaceae, Gunneraceae, Haloragaceae, Heliconiaceae, Hydrangeaceae, Hydrocharitaceae, Hymenophyllaceae, Hypericaceae, Hypoxidaceae, Iridaceae, Isoetaceae, Juglandaceae, Juncaceae, Juncaginaceae, Lamiaceae, Lardizabalaceae, Lauraceae, Lecythidaceae, Lentibulariaceae, Liliaceae, Linaceae, Linderniaceae, Lycopodiaceae, Lythraceae, Magnoliaceae, Malpighiaceae, Malvaceae, Marantaceae, Marsileaceae, Martyniaceae, Melanthiaceae, Melastomataceae, Meliaceae, Menispermaceae, Menyanthaceae, Molluginaceae, Moraceae, Moringaceae, Musaceae, Myricaceae, Myrtaceae, Nartheciaceae, Nelumbonaceae, Nephrolepidaceae, Nitrariaceae, Nyctaginaceae, Nymphaeaceae, Oleaceae, Onagraceae, Ophioglossaceae, Orchidaceae, Orobanchaceae, Osmundaceae, Oxalidaceae, Paeoniaceae, Pandanaceae, Papaveraceae, Passifloraceae, Paulowniaceae, Phrymaceae, Phyllanthaceae, Phytolaccaceae, Pinaceae, Piperaceae, Pittosporaceae, Plantaginaceae, Platanaceae, Plumbaginaceae, Poaceae, Polemoniaceae, Polygalaceae, Polygonaceae, Polypodiaceae, Pontederiaceae, Portulacaceae, Posidoniaceae, Potamogetonaceae, Primulaceae, Proteaceae, Psilotaceae, Pteridaceae, Ranunculaceae, Resedaceae, Rhamnaceae, Rosaceae, Rubiaceae, Ruppiaceae, Rutaceae, Salicaceae, Salviniaceae, Santalaceae, Sapindaceae, Sapotaceae, Sarraceniaceae, Saxifragaceae, Scheuchzeriaceae, Schizaeaceae, Scrophulariaceae, Selaginellaceae, Simaroubaceae, Smilacaceae, Solanaceae, Staphyleaceae, Strelitziaceae, Styracaceae, Tamaricaceae, Taxaceae, Theaceae, Thelypteridaceae, Thymelaeaceae, Tofieldiaceae, Tropaeolaceae, Typhaceae, Ulmaceae, Urticaceae, Verbenaceae, Violaceae, Vitaceae, Woodsiaceae, Xanthorrhoeaceae, Zingiberaceae, Zosteraceae, Zygophyllaceae

## Spatial coverage

### General geographic description

This national dataset collates species occurrences from metropolitan France and Reunion Island. Four different climates are covered in the metropolitan area: oceanic, continental, mediterranean and alpine. Reunion Island is an overseas department and region of France, located in the southwest Indian Ocean, in the Mascareignes archipelago, to the east of Madagascar. The island has a tropical climate.

The records documented in the dataset are distributed across 21 (out of a total of 27) administrative regions of France: Aquitaine, Auvergne, Bourgogne, Bretagne, Centre, Champagne-Ardenne, Corse, Franche-Comté, Ile-de-France, Languedoc-Roussillon, Limousin, Midi-Pyrénées, Nord-Pas-de-Calais, Basse-Normandie, Haute-Normandie, Pays de la Loire, Picardie, Poitou-Charentes, Provence-Alpes-Côte d’Azur, La Réunion, Rhône-Alpes.

This geographical zone is characterized by a large range in altitude, from 0 to 4,810 m above sea level, and extends over an area of 516,499 km² representing 74% of Metropolitan France, its overseas departments and other territories.

## Geographical method

### Reference grids

In France, the Muséum National d’Histoire Naturelle (MNHN) recommends the use of standardized grids for species distribution maps. The grid reference of MNHN is defined according to the French official map projection systems: Lambert 93 in Metropolitan France (grid name “L93_10X10”) and UTM 40 S in La Reunion Island (grid name “grille_10km_ZEE_974”).

Lambert-93 (EPSG 2154) is a Lambert conic projection using RGF93 geodetic system (compatible with WGS84) and defined by two reference parallels: 44°N and 49°N. The central meridian, 3°E, is the Greenwich meridian and the latitude of origin is 46°30'N. Prime coordinates are 700,000 meters and 6,600,000 meters.

The Universal Transverse Mercator Projection 40 South (UTM 40S) is an adaptation of the standard Mercator projection. This is a cylindrical and conformal projection using RGR92 geodetic system (compatible with WGS84). The central meridian is 57°E and the latitude of origin is 0. Prime coordinates are 500,000 meters and 10,000,000 meters.

Grids are composed of cells of 10 km by 10 km. Continental and maritime metropolitan France (954, 500 sq.km) is divided into 9546 cells and La Reunion Island terrestrial area (2, 512 sq.km) is divided into 34 cells (information on maritime area is not given as it is disproportionate in relation to terrestrial one).

All SIFlore records are georeferenced through the code of the corresponding grid square.

French municipalities repository: The official geographic boundaries of the municipalities and associated data (BD CARTO®) were provided by the National Geographic Institute (IGN) based on the National Institute of Statistics and Economic Studies (Insee) database. Each municipality is referenced by an official geographic code (code Insee) and its name. The records in SIFlore are georeferenced through the code of the corresponding municipality.

The BD CARTO® version originally used for data aggregation was published in 2011.

The current version is BD CARTO® 3.1 which was revised in 2013.

### Coordinates

Metropolitan France : 40°0'0"N – 52°0'0"N Latitude; 07°0'0"W – 12°0'0"W Longitude.

La Reunion Island : 20°52'0"S – 21°24'0"S Latitude; 55°10'0"E – 55°50'0"E Longitude.

### Temporal coverage

**Figure 2.**
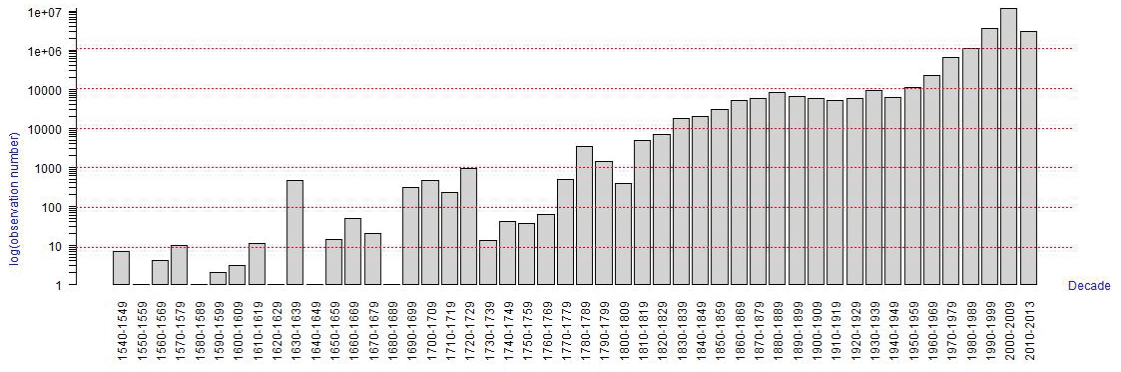
Temporal distribution of records by decade (shown on logarithmic scale).

The oldest record in the dataset is from the year 1545 and the most recent records are from 2014. Most records (69.6%) were obtained after 2000 (Figure 2).

Records for which the date of collection is unknown are registered with the year 1500.

## Project description

**Title**: «*A dataset on vascular plant distribution covering five centuries of knowledge in France:Results of a collaborative project coordinated by the Federation of the National Botanical Conservatories*» is the outcome of a collaboration between 11 Conservatoires botaniques nationaux (CBN) and their Federation (FCBN): (a) Conservatoire Botanique National Alpin, Domaine de Charance 05000 Gap, France (b) Conservatoire Botanique National de Bailleul, Hameau des Haendries 59270 Bailleul, France (c) Conservatoire Botanique National du Bassin Parisien, Muséum national d’Histoire naturelle, 61, rue Buffon 75005 Paris, France (d) Conservatoire National Botanique de Brest, 52, allée du Bot 29200 Brest, France (e) Conservatoire National Botanique de Corse, Office de l’environnement de la Corse, 14, avenue Jean Nicoli 20250 Corte, France (f) Conservatoire Botanique National de Franche-Comté, Maison de l’environnement de Franche-Comté, 7, rue Voirin

25000 Besançon, France (g) Conservatoire Botanique National de Mascarin, 2, rue du Père Georges

Les colimaçons 97436 Saint-Leu, Ile-de-La-Réunion, France (h) Conservatoire Botanique National du Massif Central, Le Bourg 43230 Chavaniac-Lafayette, France (i) Conservatoire Botanique National méditerranéen de Porquerolles, 34, avenue Gambetta 83400 Hyères, France (j) Conservatoire Botanique National des Pyrénées et de Midi Pyrénées, Vallon de Salut - BP 315- 65203 Bagnères-de-Bigorre Cedex, France (k) Conservatoire Botanique National Sud-Atlantique, Domaine de Certes Graveyron 33980 Audenge, France

### Personnel

Philippe Antonetti (Content provider, Curator), Gilles Bailly (Data manager), Christophe Bougault (Data manager), Vincent Boullet (Author, Content provider, Curator), Gregory Caze (Content provider, Curator), Gilles Corriol (Content provider, Curator), Maëlle Decherf (Project manager), Alain Delage (Content provider, Curator), Alexis Desse (Data manager, Curator), Bruno Dutrève (Author, Team Coordinator), Yorick Ferrez (Content provider, Curator), Sébastien Filoche (Content provider, Curator), Romain Gaspard (Project manager, Data manager), Olivier Gavotto (Data manager), Jean-Michel Genis (Data manager), Johan Gourvil (Author, Project manager, Curator), Julien Geslin (Content provider, Curator), Dominique Guyader (Data manager), Julien Guyonneau (Data manager, Curator), Elodie Hamdi (Data manager), Anaïs Just (Author, Project manager, Data manager, Metadata Provider), Jean-Brieuc Lehebel-Peron (Data manager), Sylvie Magnanon (Content provider, Curator), Jérôme Millet (Author, Team Coordinator), Thomas Milon (Author, Project manager, Data manager), James Molina (Content provider, Curator), Virgile Noble (Content provider, Curator), Gilles Pache (Content provider, Curator), Frédéric Picot (Content provider, Curator), Anne Plu (Data manager), Thierry Vergne (Data manager), Paula Spinosi (Data manager), Benoit Toussaint (Content provider, Curator).

### Funding

French Ministry of Ecology and the network of the CBN

## Methods

### Data collection

Primary data are collected by both professional (from CBN and other organisations) and volunteer. As data originate from various sources (field inventories, scientific literature and herbaria), this task involves different trades such as botanists and archivists. Data are entered into CBN databases and then follow a validation process. To insure dataset homogeneity, data records are extracted from CBN databases and provided to the national system in a simplified format that is compatible with the SINP format (edited by MNHN). This format is also based on the Data Specification on Species distribution, produced by the INSPIRE Thematic Working Group *Species distribution* (http://inspire.ec.europa.eu/).

For each observation, key variables are recorded, such as a unique code for the record, valid scientific name of the taxon and its identifier in the French national taxonomic repository (TAXREF). Additional details including observation and data transmission dates, the collector name, the source basis of record name and geospatial information, including municipality name and code, and the square code of the national grid, are also provided. The grid system used for species inventories in France is defined by the Muséum National d’Histoire Naturelle: this is a grid composed of cells of 10km by 10km. Information about municipalities are collected from the Insee national repository. When available, additional information is collected such as bibliographic and herbarium references or primary source of the data record.

The evolution of the dataset is described in the Database history section.

Once standardized, data are checked for consistency before being incorporated into the Postgresl/Postgis national database by using Talend Open Studio for Data Integration. At the end of the process, data are posted on the FCBN geoportal.

**Sampling description**: Most records originate from field inventories (88.7%), with other records identified in scientific literature (11%) and in herbaria (0.2%). The protocols for collection vary over time and between collection sites, but also in response to other projects launched. However, in this first version, SIFlore does not include information on the data collection procedures. It is expected that a simplified description of the different protocols used by each CBN will be provided in the near future.

**Figure 3.**
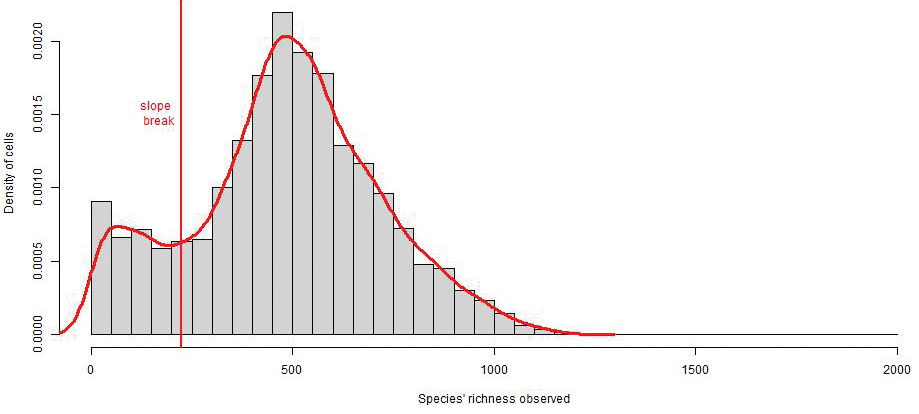
Density of cells by richness of observed species: looking at the distribution within the dataset, it appears that cells with less than 250 distinct species recorded are over-represented.

**Figure 4.**
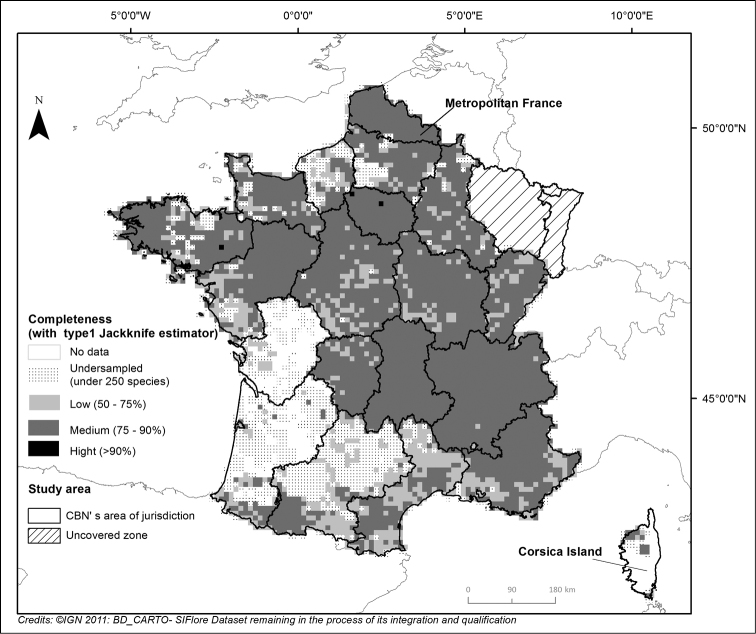
Dataset completeness for Metropolitan France according to the Jackknife 1 estimator (data from 1990 to 2013). The number of records in each cell was used as an estimator of the sampling effort. The ratio between the observed and estimated richness of species measures the completeness of inventory in each surveyed cell (Vallet et al. 2012).

Nevertheless, according to Vallet et al. (2012), it is possible to assess survey completeness of regional floristic inventories despite heterogeneous sources and protocols, through the use of the Jackknife 1, a non-parametric estimator. This estimator was calculated for each 10km by 10km cell of the French map, aiming to mitigate sampling bias as effectively as possible. Thus, occurrences were all generalized to the rank of species (including all infraspecies into this one rank) and only recent data were taken in account (from 1990). Cells with less than 250 species were excluded from the analyses, as they were considered as undersampled and therefore over represented (Figure 3). The resulting map (Figure 4) should allow users to interpret their results appropriately.

It has to be noted that the completeness of La Reunion Island survey could not be assured in this work. Indeed, as mentioned above, in 2012, at the time of working on the 1^st^ version of SIFlore, there had been a lack of La Reunion taxa in the French national taxonomic referential for fauna, flora and fungi, TAXREF V5. As the analysis only takes into account the species which correspond across the two taxonomic guidelines (ITR and TAXREF v5), all of the La Reunion cells had been undersampled, according to Jackknife calculation requirements.

**Quality control description**: Quality control is implemented at different levels, under different responsibilities, throughout the data collection and validation process. Following digitalization, the dataset is first checked by regional data administrators, in order to ensure compliance with the survey protocols. The records are individually reviewed according to specified criteria, including the accuracy of the scientific name and the correctness of the geographical position entered, according to known chorology.

At the national level, a second step is carried out to ensure conformity of the data to the national standards, before compilation. Non-compliant data are rejected and an audit report is sent to the data provider. FCBN is currently working on an additional quality control step in order to ensure global coherence and to provide a relevance score for the distribution map of each species.

## Dataset

### Dataset history

In 1975, the Botanical Conservatory of Brest was created, with the support of the Ministry of Environment. This was the first establishment in the world entirely devoted to flora conservation. In 1988, the label “conservatoire botanique national” was legally recognized in France. There are currently eleven national botanical conservatories (CBN) in France.

As they are mandated to share their expertise to the national and local authorities, CBN have built knowledge databases in which information is structured to allow data sharing.

CBN have operational and managerial autonomy. Consequently, their databases differ in terms of structural design and the information contained. Nevertheless, there is an urgent need to provide information on flora distribution at the national level and, in particular, to define more clearly the flora conservation priorities. Furthermore, FCBN is involved in establishing the IUCN Red List of Threatened Flora at the national level, and in evaluating the conservation status of natural habitats and wild flora, according to the *Council Directive 92/43/EEC of 21 May 1992 on the conservation of natural habitats and of wild fauna and flora*. For these reasons, it was decided to pool all CBN flora records and also to apply a subsidiarity relationship between the FCBN and CBN. This means that data must only be handled by national botanical conservatories, and that the federation may only facilitate networking and ensure data aggregation and management, without altering the records in any way.

In 2010, a working group was created to implement this project. It includes botanists and data managers from across the CBN, and project facilitators. Due to the heterogeneity of the CBN databases, data cannot easily and quickly be aggregated unless determining a database exchange standard. As such, a standardized format was proposed and, in 2011, 10 taxa were selected for a first trial run. Based on this pilot, the data standard was refined. In January 2013, the flora regional dataset aggregation was officially launched. Data were transmitted through various channels under the scenarios prepared by the working group. Data were then compiled and integrated into a Postgresql/Postgis database using an extract, transform and load system (ETL). In May 2013, 18 million data records were aggregated. In January 2014, an additional 3 million records were integrated. Meanwhile, a geoportal was developed to respond to the needs of the CBN and their partners for improving their understanding of flora distribution, as well as allowing the CBN to share its expertise with the public. The portal was finally published on the FCBN’s website in February 2014.

### Dataset description

The SIFlore dataset is a custom-made SQL view of the global database hosted by the FCBN. Only tracheophyta data are shown. In the current version of the database, the key information provided for each record includes: unique identifier of the data record in SIFlore, identifier that points to the data in the original database file, source institution and database identifier, scientific name, valid identifier in the French national taxonomic referential (TAXREF), taxon rank, location of sighting (grid cell and municipality code), date that the flora was sighted and name of the data collector.

### Dataset preview

**Object name**: SIFlore, a dataset of geographical distribution of vascular plants covering five centuries of knowledge in France: Results of a collaborative project coordinated by the Federation of the National Botanical Conservatories

**Character encoding**: UTF-8

**Format name**: Darwin Core Archive Format

**Format version**: 1.0

**Distribution**: http://www.gbif.org/dataset/75956ee6-1a2b-4fa3-b3e8-ccda64ce6c2d

**Publication date of data**: 2014-02-10

**Language**: French

**Licenses of use**: This “SIFlore, a dataset of geographical distribution of vascular plants covering five centuries of knowledge in France: Results of a collaborative project coordinated by the Federation of the National Botanical Conservatories” is made available under the Creative Commons Attribution Non Commercial (CC BY-NC-SA 2.0 FR)

**Metadata language**: English

**Date of metadata creation**: 2014-06-26

**Hierarchy level**: Dataset

## Supplementary materials

Supplementary material 1 Numerical appendix Data type: dataset Explanation note: A shapefile representing the dataset completeness (based on the Jackknife 1, a non-parametric estimator) on a grid of 10 km by 10 km cells. The number of records in each cell was used as an estimator of the sampling effort. The ratio between the observed and estimated richness of species measures the completeness of the inventory in each surveyed cell (Vallet et al. 2012). This dataset is made available under the Open Database License (http://opendatacommons.org/licenses/odbl/1.0/). The Open Database License (ODbL) is a license agreement intended to allow users to freely share, modify, and use this Dataset while maintaining this same freedom for others, provided that the original source and author(s) are credited.Anaïs Just, Johan Gourvil, Jérôme Millet, Vincent Boullet, Thomas Milon, Isabelle Mandon, Bruno Dutrève
